# Standardized Order Sets Do Not Eliminate Racial or Ethnic Inequities in Postpartum Pain Management

**DOI:** 10.1089/heq.2022.0180

**Published:** 2023-10-12

**Authors:** Celeste A. Green, Jasmine D. Johnson, Christine McKenzie, Alison M. Stuebe

**Affiliations:** ^1^ Department of Obstetrics and Gynecology, University of Chapel Hill, Chapel Hill, North Carolina, USA.; ^2^ Division of Maternal Fetal Medicine, Department of Obstetrics and Gynecology, Indiana University, Indianapolis, Indiana, USA.; ^3^ Department of Anesthesiology, University of North Carolina Chapel Hill, Chapel Hill, North Carolina, USA.; ^4^ Division of Maternal Fetal Medicine, Department of Obstetrics and Gynecology, University of North Carolina Chapel Hill, Chapel Hill, North Carolina, USA.

**Keywords:** acetaminophen, disparity, ERAS, narcotics, NSAIDs, opioids

## Abstract

**Objective:**

To quantify the extent to which a standardized pain management order set reduced racial and ethnic inequities in post-cesarean pain evaluation and management.

**Methods:**

We conducted a retrospective cohort study to quantify racial and ethnic differences in pain evaluation and management before (July 2014–June 2016) and after implementation of a standardized post-cesarean order set (March 2017–February 2018). Electronic medical records were queried for pain scores >7/10, number of pain assessments, and opioid, nonsteroidal anti-inflammatory drug (NSAID), and acetaminophen doses. Outcomes were grouped into 0 to <24 and 24–48 h postpartum, and stratified by race/ethnicity (Hispanic, non-Hispanic Black [NHB], non-Hispanic White [NHW], Asian, and other), as documented in the electronic health record. Analyses included logistic regression for the categorical outcome of pain score >7 (severe pain), and linear regression, with propensity score adjustment. Main effect and interaction terms were used to calculate the difference-in-difference in pain process and outcome measures between the baseline and follow-up periods.

**Results:**

After order set implementation (*N*=888), severe pain remained more common among NHB patients (% pain scores >7 NHW vs. NHB 0 to <24 h: 22% vs. 33%, *p*=0.003; 24–48 h: 26% vs. 40%, *p*<0.001). Among all patients, pain management processes changed after implementation of the order set, with overall fewer assessments, less Opioids, and more nonopioid analgesics. However, racial and ethnic inequities in a number of assessments and in treatment were unchanged (all *p* for interaction >0.05), with the exception of a modest increase in NSAID doses 24–48 h postpartum for Hispanic patients.

**Conclusion:**

A standardized pain management order set reduced overall postpartum opioid use, but did not reduce racial and ethnic disparities in pain evaluation and management. Future work should investigate racial equity-focused education and interventions designed to eliminate disparities in pain management.

## Introduction

Previous studies in multiple medical specialties have demonstrated disparities in the experience, diagnosis, and treatment of pain between patients of different races and ethnicities.^1^ Few have explored effective ways to reduce these disparities. In prior work, we found statistically significant differences by race and ethnicity in the assessment and treatment of pain following cesarean delivery at our institution.^2^ In the first 48 h postpartum, non-Hispanic White (NHW) patients received more pain assessments, more nonsteroidal anti-inflammatory drugs (NSAIDs) and acetaminophen, and more opioids compared with non-Hispanic Black (NHB) and Hispanic patients. These disparities were present even though NHB and Hispanic patients reported higher pain scores than did NHW or Asian patients; this trend has also been demonstrated at other institutions.^3^

Standardization is a recommended strategy to reduce undesired variation in practice.^4–6^ In October 2016, a new, mandatory protocol was implemented at our institution for pain management after cesarean delivery with the goal of reducing postpartum opioid use. In the new standardized protocol, the anesthesiology team took responsibility for entering a single postpartum pain management order set for all patients. Before the new protocol, post-cesarean pain management orders were usually placed by an Obstetrics service resident using their own personalized order sets.

We sought to determine the extent to which standardization of the post-cesarean pain management order set decreased racial and ethnic inequities demonstrated in our prior work. We hypothesized that this new protocol would not eliminate the previously observed inequities.

## Methods

In November 2017, a standardized order set for post-cesarean pain management was instituted. This is a retrospective cohort study of patients who had live births through cesarean delivery at University of North Carolina (UNC)-Chapel Hill between March 1, 2017, and February 28, 2018. The pain assessment and treatment data were compared with the baseline period, July 1, 2014, to June 30, 2016, which was the timeframe of our prior study demonstrating inequity in assessment and management of pain.

Use of the new order set was universally accepted and became part of our Labor and Delivery unit's standard operating procedure. The 5-month delay between order set introduction and the start date of our study period allowed a washout period for the residents, attendings, and nursing staff to familiarize themselves with the new standard of care. Placed by the anesthesiology team at the end of every cesarean delivery, the standardized set included the following: acetaminophen 1000 mg once post-operatively, followed by 650 mg scheduled every 6 h; intravenous ketorolac 30 mg or oral ibuprofen 600 mg scheduled every 6 h for the first 24 h, followed by oral ibuprofen 600 mg scheduled every 6 h; and oral oxycodone 5–10 mg as needed for pain score >7 (severe pain).

The order set did not specify frequency of pain assessment. The hospital's nursing policy stipulates that pain be assessed at least once per shift, and as needed per patient request; it requires reassessment of pain within 1 h of any intervention for pain treatment, including as needed pain medications. The nursing ratio is one to every four mother/infant dyads.

Data were extracted from the UNC at Chapel Hill electronic health record (EHR) through the UNC Perinatal Research Service Center and the UNC Epic Data Warehouse for Health. Sociodemographic data for the study cohort include maternal age, marital status, insurance at delivery, primary spoken language, site of prenatal care, and self-reported race and ethnicity, as documented in the EHR. The racial and ethnic groups included NHW (referent group), NHB, Hispanic ethnicity, Asian, and Other, which included those patients with unknown race, more than one documented race, or race documented as Native American or Alaskan Native.

Clinical data extracted from the EHR include time and date of delivery, maternal body mass index (BMI) (either self-reported or documented at earliest time from 90 days before the last menstrual period through delivery), parity, history of prior cesarean delivery, classical cesarean delivery, gestational age at delivery, and whether an infant was admitted to the neonatal intensive care unit (NICU).

We excluded from our sample those who underwent cesarean hysterectomies and general anesthesia during delivery, received patient-controlled analgesia or infusion of any intravenous narcotic, had a history of chronic opioid use (indicated by active use of buprenorphine and methadone in the hospital admission or at least two opioid prescriptions in the prenatal period), or for whom the above-listed clinical or demographic information was missing.

Pain scores were indexed 0 to <24 h or 24 to 48 h from delivery to match prior study parameters. Opioid medications administered were tabulated in 5 mg oxycodone tablet equivalents (OTEs) for all opioid medications, including morphine, hydromorphone, codeine, and oxymorphone. The predominant opioid administered was a 5 mg oxycodone tablet, as dictated in the order set. Doses of acetaminophen and NSAIDs such as ibuprofen and ketorolac were also tabulated by 0 to <24 h and 24 to 48 h postpartum.

For each racial or ethnic group, we calculated the number of pain scores >7 (severe pain), and total OTEs, NSAID, and acetaminophen doses administered, in the 0 to <24 and 24 to 48 h postpartum periods. Logistic regression was used to analyze the proportion of patients with severe pain scores (>7), and linear regression was used to compare continuous measures by patient race and ethnicity. Following the methodology of our prior study,^2^ we created propensity scores using multinomial regression to calculate the probability that a participant belonged to each of the five race-ethnicity groups as a function of maternal age, BMI, gestational age at delivery, nulliparity, primary versus repeat cesarean delivery, classical versus not classical hysterotomy, and infant admission to the NICU. These propensity scores were used in adjusted regression models.

The purpose of our analysis was to determine whether implementation of a standardized order set narrowed the previously described gap between pain measures in NHW patients and NHB or Hispanic patients. We therefore performed a difference-in-difference calculation to answer, “Did implementation reduce the previously identified inequities in experience of severe pain, number of assessments of pain, and administration of opioid and nonopioid analgesia?” To measure overall change in practice, we used an “intervention x race/ethnicity” interaction term to answer our primary question. For ease of interpretation, we presented least square means for each measure before and after the intervention.

Data analyses were performed using SAS 9.4 (SAS Institute Inc., Cary, NC). The study received approval in accordance with the study center's Institutional Review Board.

## Results

A total of 888 patients gave birth by cesarean during the study period and met inclusion criteria. In the follow-up period, NHB patients (*N*=232) comprised 26.1% of the study population, and most NHB patients were in the 25–29-year age group (29.7%). Asian (*N*=37), Hispanic (*N*=164), and patients of other races (*N*=75) were older, with 97/276 patients across these groups 35 years of age or older.

NHW patients (total *N*=380) were also relatively older, with the largest proportion in the 30–34-year age group (*N*=135, 35.5%). NHB patients, Hispanic patients, and patients classified as Other were more likely to have Medicaid insurance at delivery (51.7%, 76.2%, and 40%, respectively). NHW patients were more likely to be nulliparous. Most patients in each group received their prenatal care at our institution, except for Hispanic patients, among whom 55.5% received care elsewhere. The predominant primary spoken language was English, but 29.7% of Asian patients had a primary language other than Spanish or English.

Hispanic and NHB parturients were more likely than NHW to have had a previous cesarean delivery. Most infants were born at term, with 54.6% of all infants born at or after 39 weeks of gestation (*N*=485.) Of those who delivered preterm, (*N*=173, 19.5%), NHB patients had the highest rate of preterm birth in the study period, with 46 infants (26.6%) born at <37 weeks of gestation (Table 1).

**Table 1.** Study Cohort Demographics and Clinical Characteristics

	Asian, ***n*** (%)	NHB, ***n*** (%)	Hispanic, ***n*** (%)	Other race, ***n*** (%)	NHW, ***n*** (%)	***p***
Total	37	232	164	75	380	
Age, years						<0.01
<20	0 (0.0)	<10 (2.2)	<10 (4.9)	<10 (8.0)	<10 (1.3)	
20–24	<10 (5.4)	48 (20.7)	24 (14.6)	<10 (12.0)	24 (6.3)	
25–29	<10 (5.4)	69 (29.7)	29 (17.7)	18 (24.0)	88 (23.2)	
30–34	12 (32.4)	52 (22.4)	50 (30.5)	19 (25.3)	135 (35.5)	
35+	21 (56.8)	58 (25.0)	53 (32.3)	23 (30.7)	128 (33.7)	
Marital status						<0.01
Unmarried	<10 (2.7)	131 (56.5)	76 (46.3)	23 (30.7)	75 (19.7)	
Married	35 (94.6)	89 (38.4)	74 (45.1)	24 (32.0)	281 (73.9)	
Missing	<10 (2.7)	12 (5.2)	14 (8.5)	28 (37.3)	24 (6.3)	
Primary language						<0.01
English	26 (70.3)	229 (98.7)	66 (40.2)	66 (88.0)	379 (99.7)	
Spanish	0 (0.0)	0 (0.0)	97 (59.1)	<10 (4.0)	0 (0.0)	
Other	11 (29.7)	<10 (1.3)	<10 (0.6)	<10 (8.0)	<10 (0.3)	
Insurance at delivery						<0.01
Private	31 (83.8)	58 (25.0)	18 (11.0)	36 (48.0)	245 (64.5)	
Private and Medicaid	<10 (8.1)	52 (22.4)	11 (6.7)	<10 (4.0)	38 (10.0)	
Medicaid	<10 (8.1)	120 (51.7)	125 (76.2)	30 (40.0)	93 (24.5)	
Unknown	0 (0.0)	<10 (0.9)	10 (6.1)	<10 (8.0)	<10 (1.1)	
Prenatal care at institution						<0.01
Yes	32 (86.5)	165 (71.1)	73 (44.5)	40 (53.3)	294 (77.4)	
BMI (kg/m^2^)						<0.01
<25	24 (64.9)	45 (19.4)	31 (18.9)	24 (32.0)	140 (36.8)	
25–29.9	11 (29.7)	52 (22.4)	57 (34.8)	19 (25.3)	94 (24.7)	
≥30	2 (5.4)	135 (58.2)	76 (46.3)	32 (42.7)	146 (38.4)	
Multiparous	22 (59.5)	147 (63.4)	128 (78.0)	38 (50.7)	220 (57.9)	<0.01
Primary cesarean delivery	23 (62.2)	130 (56.0)	77 (47.0)	52 (69.3)	218 (57.4)	0.02
Low transverse incision	37 (100.0)	223 (96.1)	158 (96.3)	71 (94.7)	369 (97.1)	0.62
Gestational age, weeks						0.51
<37	<10 (13.5)	46 (19.8)	26 (15.9)	16 (21.3)	80 (21.1)	
37 to <39	10 (27.0)	73 (31.5)	42 (25.6)	17 (22.7)	88 (23.2)	
39 to <41	19 (51.4)	100 (43.1)	89 (54.3)	36 (48.0)	192 (50.5)	
>41	<10 (8.1)	13 (5.6)	<10 (4.3)	<10 (8.0)	20 (5.3)	
Infant ever in NICU	<10 (21.6)	71 (30.6)	28 (17.1)	21 (28.0)	101 (26.6)	0.04

Chi square *p*-values.

BMI, body mass index; NHB, non-Hispanic Black; NHW, non-Hispanic White; NICU, neonatal intensive care unit.

**FIG. 1.**
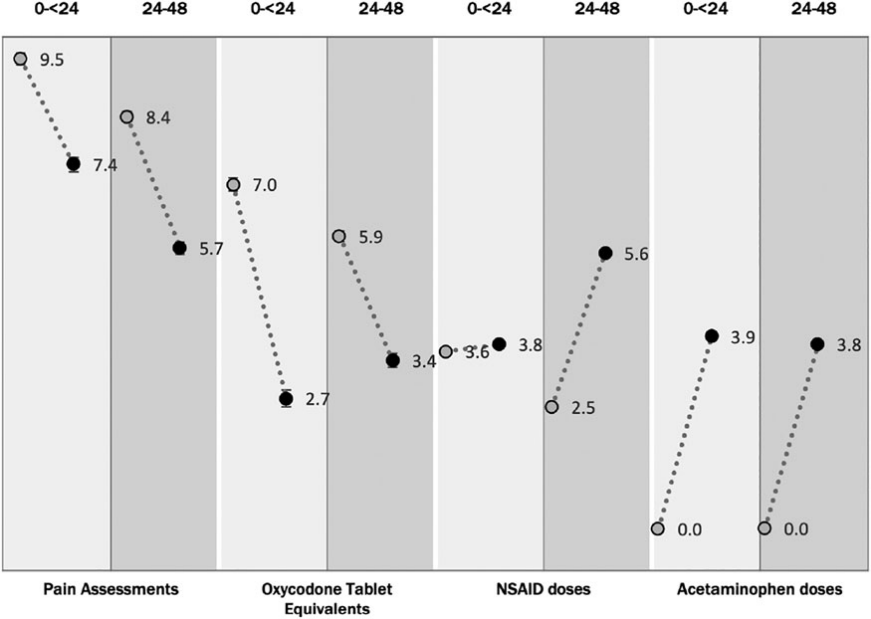
Overall change in pain measures from pre-implementation to post-implementation. NSAID, nonsteroidal anti-inflammatory drug.

**FIG. 2.**
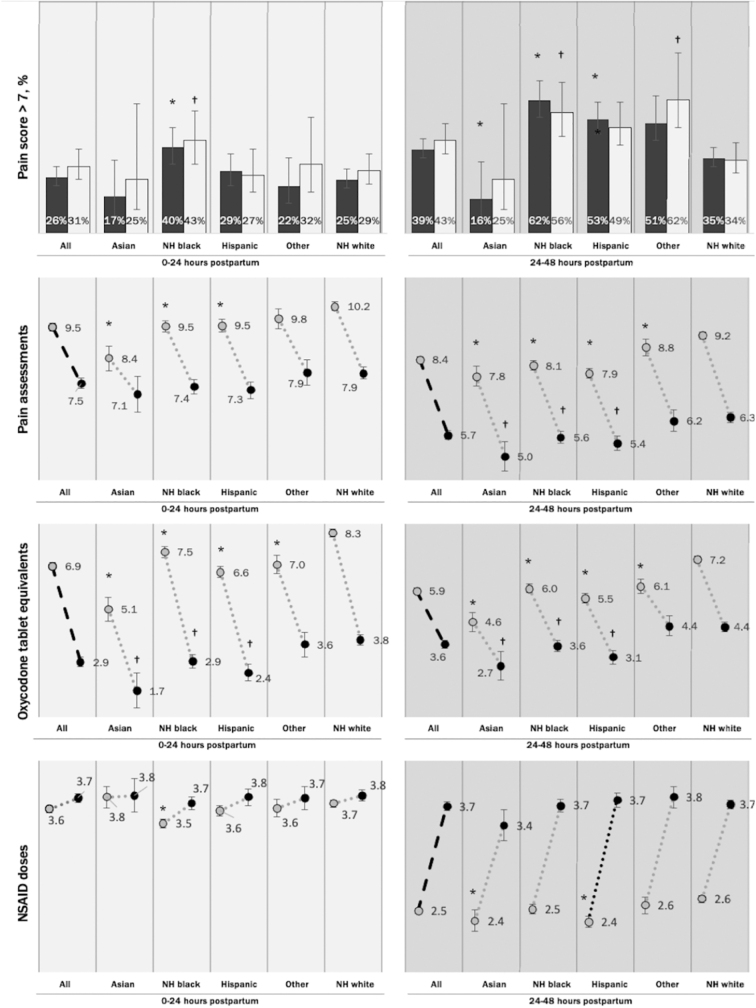
Difference-in-difference pre-implementation and post-implementation for pain measures, stratified by race and ethnicity compared to NHW referent group. *p < 0.05 for comparison of pre-intervention with pre-intervention NHW. ^†^*p* < 0.05 for comparison of post-intervention with post-intervention NHW. NHB, non-Hispanic Black; NHW, non-Hispanic White.

As intended by the new order set, compared with the baseline population (*N*=1701), the post-implementation population (*N*=888) received less OTEs (0 to <24 h: 7.0 vs. 2.7, *p*<0.0001; and 24–48 h: 5.9 vs. 3.4, *p*<0.0001), more NSAIDs at 24–48 h (0 to <24 h: 3.6 vs. 3.8, *p*=0.17; 24–48 h: 2.5 vs. 5.6, *p*<0.0001), and more acetaminophen (0 to <24 h: 0.0 vs. 3.9, *p*<0.0001; 24–48 h: 0.0 vs. 3.8, *p*<0.0001; Fig. 1). Although the order set implementation changed overall pain management practices, it did not affect inequities (Fig. 2).

In the post-implementation period, compared with NHW patients, NHB patients continued to be more likely to experience pain scores >7 (propensity score adjusted probability, 0 to <24 h: 43% vs. 29%; 24 to <48 h: 56% vs. 34%, *p*<0.05). NHB, Hispanic, and Asian patients underwent fewer pain assessments at 24 to 48 h (NHB 5.6, Hispanic 5.4, Asian 5.0, vs. NHW 6.3, *p*<0.05 for each comparison), and received fewer OTEs (0 to <24 h: NHB 2.9, Hispanic 2.4 and Asian 1.7 vs. NHW 3.8; 24 to <48 h: NHB 3.6, Hispanic 3.1, and Asian 2.7, vs. NHW 4.4, *p*<0.05 for each comparison).

With respect to NSAID dosing, baseline to follow-up changes in number of doses, illustrated by the slope of lines in Figure 2, did not differ by race/ethnicity, except for NSAID doses for Hispanic patients at 24 to 48 h. These increased by 0.4 doses more (95% confidence interval: 0.1–0.7, *p*<0.01) for Hispanic patients than for NHW patients. There was no difference in acetaminophen dosing by race-ethnicity from pre-implementation to post-implementation (data not shown).

## Discussion

While standardization of protocols has been promoted in other obstetric contexts—such as postpartum hemorrhage^7^—as a strategy to reduce inequities, we found that for postpartum pain management at our institution, “equal” treatment did not produce equitable outcomes. As previously reported,^8^ a standardized order set reduced postpartum opioid usage, consistent with other literature showing that standard order sets for cesarean delivery patients decrease opioid usage and lead to shorter hospital stays.^9,10^ This is the first study, to our knowledge, that looked specifically at such order sets' impact on racial and ethnic disparities in obstetric patients. Our findings underscore the importance of designing interventions specifically to address racial and ethnic inequities, and of measuring processes and outcomes stratified by race and ethnicity, as advocated for in the Alliance for Innovation on Maternal Health's Reduction of Peripartum Racial/Ethnic Disparities bundle.^11^

Maternity care bundles are a relatively recent application of care bundles as a strategy to improve care.^12^ One successful example showed reduction in severe maternal morbidity (SMM) from postpartum hemorrhage for every racial and ethnic group after implementation of a standard protocol across 99 different California hospitals. The reduction in morbidity was greatest for NHB patients, among whom the rate of SMM was highest before the intervention. Effectiveness in that study may have been attributed to the nationally recognized standards and outcome measures for postpartum hemorrhage, which allowed for a clearly defined intervention to be developed. As those authors concluded, the variable effect on disparity reduction seen in quality improvement studies may stem from the fact that the “focus of the intervention alone does not sufficiently address the primary sources of racial/ethnic disparities.”^7^

That standardized orders did not address inequities in our study calls in to question how implicit bias, institutionalized racism, and discrimination influence health outcomes. Many adverse pregnancy outcomes—from rates of preterm birth^13^ to maternal deaths^14,15^—disproportionately affect patients of color, and cannot be explained by differences in levels of income, insurance status, or access to prenatal care.^16^

Limitations of our study include its retrospective nature, which precludes the ability to survey patients' satisfaction with their post-operative pain management, as well as factors that might influence decisions to accept or decline pharmacologic treatment for pain. Such information will be needed to design more equitable pain treatment strategies. We suggest that additional research explore adjunctive approaches to pharmacologic pain treatment, while incorporating patient input on which cultural and social factors influence^17^ patients' experiences of pain. A significant limitation of order set implementation, particularly regarding addressing systemic and implicit racism, is that the order set does not incorporate any additional assessment than the once-per-shift assessment per the postpartum floor nursing protocol.

Strengths of our study include consistency with the baseline study's design, strengthening the reliability of our reported results.

## Health Equity Implications

On a population level, the impact of disparities in post-cesarean delivery pain experience and treatment is further compounded by the fact that NHB patients have a higher rate of cesarean delivery than NHW patients. In 2019, 35.9% of NHB patients underwent cesarean delivery, compared with 31.3% of Hispanic patients and 30.7% of NHW patients.^13^ As NHB patients already bear a disproportionate share of SMM, having more than one in three NHB births being through cesarean adds even more undue burden for this population. More equitable post-operative pain control is urgently needed to improve the postpartum experience.

Our work suggests several future opportunities for investigation with respect to improved post-operative pain management, such as establishing more concrete guidelines for pain assessment and treatment,^1,2^ ensuring use of foreign language interpreters for each patient encounter, and^3^ defining patients' priorities and values in setting expectations for appropriate postpartum pain through qualitative studies and focus groups. Furthermore, we recommend developing protocols with the specific goal of eliminating inequity and devising patient-centered counseling and educational resources to set mutual expectations for postpartum pain and management techniques.

Most importantly, increasing public awareness about these inequities will help multidisciplinary care teams identify opportunities for improvement within their own care settings. As intentional as our institution was in designing an order set to address the ongoing opioid epidemic, we need to be as deliberate about eliminating barriers to equitable care for all.

## Abbreviations Used

BMI: body mass index
EHR: electronic health record
NHB: non-Hispanic Black
NHW: non-Hispanic White
NICU: neonatal intensive care unit
NSAID: nonsteroidal anti-inflammatory drug
OTEs: oxycodone tablet equivalents
SMM: severe maternal morbidity
UNC: University of North Carolina
